# Development of a Multiplex PCR Assay for Genotyping the Fish Pathogen *Piscirickettsia salmonis* Through Comparative Genomics

**DOI:** 10.3389/fmicb.2021.673216

**Published:** 2021-06-11

**Authors:** Adolfo Isla, J. Eduardo Martinez-Hernandez, Héctor A. Levipan, Denise Haussmann, Jaime Figueroa, Maria Cecilia Rauch, Vinicius Maracaja-Coutinho, Alejandro Yañez

**Affiliations:** ^1^Instituto de Bioquímica y Microbiología, Universidad Austral de Chile, Valdivia, Chile; ^2^Interdisciplinary Center for Aquaculture Research (INCAR), University of Concepcion, Concepción, Chile; ^3^Departamento de Ciencias Básicas, Facultad de Ciencias, Universidad Santo Tomás, Santiago, Chile; ^4^Centro de Modelamiento Molecular, Biofísica y Bioinformática – CM2B2, Facultad de Ciencias Químicas y Farmacéuticas, Universidad de Chile, Santiago, Chile; ^5^Programa de Doctorado en Genómica Integrativa, Vicerrectoría de Investigación, Universidad Mayor, Santiago, Chile; ^6^Laboratorio de Biología de Redes, Centro de Genómica y Bioinformática, Facultad de Ciencias, Universidad Mayor, Santiago, Chile; ^7^Laboratorio de Ecopatología y Nanobiomateriales, Departamento de Biología, Facultad de Ciencias Naturales y Exactas, Universidad de Playa Ancha, Valparaiso, Chile; ^8^Instituto Vandique, João Pessoa, Brazil; ^9^Beagle Bioinformatics, Santiago, Chile; ^10^Facultad de Ciencias, Universidad Austral de Chile, Valdivia, Chile

**Keywords:** Chile, genotyping, *Piscirickettsia salmonis* detection, salmonid fish, comparative genomic

## Abstract

*Piscirickettsia salmonis* is a bacterial pathogen that severely impact the aquaculture in several countries as Canada, Scotland, Ireland, Norway, and Chile. It provokes Piscirickettsiosis outbreaks in the marine phase of salmonid farming, resulting in economic losses. The monophyletic genogroup LF-89 and a divergent genogroup EM-90 are responsible for the most severe Piscirickettsiosis outbreaks in Chile. Therefore, the development of methods for quick genotyping of *P. salmonis* genogroups in field samples is vital for veterinary diagnoses and understanding the population structure of this pathogen. The present study reports the development of a multiplex PCR for genotyping LF-89 and EM-90 genogroups based on comparative genomics of 73 fully sequenced *P. salmonis* genomes. The results revealed 2,322 sequences shared between 35 LF-89 genomes, 2,280 sequences in the core-genome of 38 EM-90 genomes, and 331 and 534 accessory coding sequences each genogroup, respectively. A total of 1,801 clusters of coding sequences were shared among all tested genomes of *P. salmonis* (LF-89 and EM-90), with 253 and 291 unique sequences for LF-89 and EM-90 genogroups, respectively. The Multiplex-1 prototype was chosen for reliable genotyping because of differences in annealing temperatures and respective reaction efficiencies. This method also identified the pathogen in field samples infected with LF-89 or EM-90 strains, which is not possible with other methods currently available. Finally, the genome-based multiplex PCR protocol presented in this study is a rapid and affordable alternative to classical sequencing of PCR products and analyzing the length of restriction fragment polymorphisms.

## Introduction

*Piscirickettsia salmonis* is a facultative intracellular Gram-negative bacterium (Fryer and Hedrick, 2003; Mauel et al., 2008) and the etiological agent of Piscirickettsiosis, a systemic infection that affects all farmed salmon species (Mauel et al., 2008). In Chile, the bacterium is widely distributed and costs the salmon farming industry up to US$700 million per year (Maisey et al., 2017). In the first half of 2019, *P. salmonis* was the leading cause of mortality in farmed Atlantic salmon and rainbow trout (Sernapesca, 2020b). Consequently, 93.85% of all antibiotics used between January and June, 2020 in Chilean seawater aquaculture (Sernapesca, 2020a) were used to treat Piscirickettsiosis outbreaks. *P. salmonis* is also present in Canada (Brocklebank et al., 1993), Scotland (Grant et al., 1996), Ireland (Rodger and Drinan, 1993), and Norway (Olsen et al., 1997). *P*. *salmonis* is also present in different non-salmonid fish species, such as Blackspot grouper (*Epinephelus melanostigma*) (Chen et al., 2000), White sea bass (*Atractoscion nobilis*) (Arkush et al., 2005), and European sea bass (*Dicentrarchus labrax*) (Athanassopoulou et al., 2004; McCarthy et al., 2005). It has also been identified in different fish species in Chile (Contreras-Lynch et al., 2015).

Initially, *P. salmonis* was misclassified as a member of the order *Rickettsiales* (Cvitanich et al., 1991). However, the bacterium was reclassified as a member of the *Gammaproteobacteria* class of the phylum *Proteobacteria* following 16S rRNA sequencing (Fryer et al., 1992). Geographically diverse *P. salmonis* isolates (e.g., from Chile, Norway, Canada, Ireland, among others) mainly belong to the monophyletic group denominated LF-89 (reference strain ATCC VR-1361) or an outlier group related to the Chilean EM-90 strain (Heath et al., 2000; Saavedra et al., 2017). Additionally, Saavedra et al. (2017), analyzed 500 field isolates of *P*. *salmonis* and showed that 50% of samples were phylogenetically related to EM-90 or LF-89 isolates. They also found that genetic groups (or genogroups) were widely distributed and responsible for outbreaks of Piscirickettsiosis in salmon farms.

The existence of two genetic groups was reinforced after a comparative genomic analysis of 19 fully sequenced *P*. *salmonis* isolates (Nourdin-Galindo et al., 2017). This notion has also been experimentally supported by studies based on Multilocus Sequence Typing (MLST) and PCR amplification of 16S rRNA genes followed by restriction fragment length polymorphism (PCR-RFLP) (Isla et al., 2019; Aravena et al., 2020). Significant differences in genomic and phenotypic traits between *P*. *salmonis* genogroups highlight the need to provide alternative methodologies for genotyping. For instance, comparative analyses of core-genomes have revealed diverse genes encoding molecular functions related to antioxidant activity, transcriptional factors, translational regulation, and genogroup-specific virulence factors (Nourdin-Galindo et al., 2017). Genomic differences between genogroups grown at different culture conditions can often be reflected at the phenotypic level. For instance, EM-90-like strains grown at 22°C have a mucoid phenotype in culture plates absent in LF-89 strains (Nourdin-Galindo et al., 2017; Saavedra et al., 2017). Additionally, challenge assays in fish result in macroscopic lesions in the intestines, brain, and skeletal muscle with an EM-90-like isolate, but not an LF-89-like isolate (Rozas-Serri et al., 2017). Furthermore, fish infected with an EM-90-like strain presented an exacerbated immune response and higher cumulative mortality in a shorter time than LF-89 infections (Rozas-Serri et al., 2017).

Genomic and phenotypic differences between EM-90 and LF-89 genogroups require new tools for fast discrimination of members belonging to both genogroups in field samples and laboratory cultures. Additionally, different farmed salmonids could be a source of diverse *P. salmonis* genogroups, especially of LF-89 and EM-90 genogroups. The present study outlines a multiplex PCR procedure for use with LF-89 and EM-90 genogroups based on a comparative genomics approach which is rapid, economical, and straightforward.

## Methodology

### Datasets

This study analyzed chromosomal whole-genome sequences belonging to 73 Chilean *P. salmonis* strains. Sequences were downloaded from the National Center for Biotechnology Information (NCBI) database (Sayers et al., 2020) in September 2020 (Supplementary Table 1). Excluding plasmid sequences and draft genomes (contig and/or scaffold level).

### Prediction of Protein-Encoding Genes in *P. salmonis* Genomes

**FIGURE 1:**
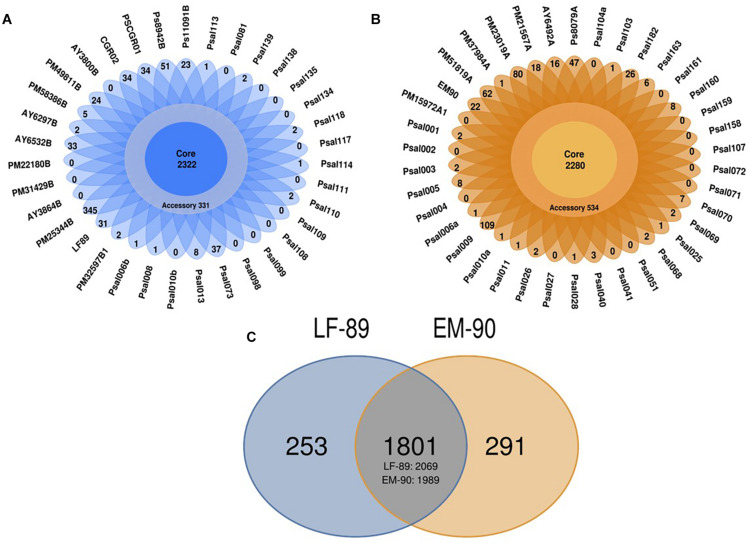
*In silico* identification of core-genome in 73 genomes of *P. salmonis*. **(A, B)** Core and Accessory genes in LF-89 and EM-90 genomes, respectively. **(C)** Ven-diagram of unique and shared sequences between LF-89 and EM-90 genomes. The intersection corresponds to shared gene clusters, and the numbers under the intersection correspond to the total number of genes counted.

Prodigal was used to predict the complete repertoire of protein-encoding genes available in all *P. salmonis* genomes (Hyatt et al., 2010) with default parameters to obtain nucleotide and amino acid sequences for each genome. Predicted open reading frames (ORFs) were functionally annotated using an automated in-house pipeline using Eggnog-mapper version 2.0.1-14-gbf04860, based on EggNOG 4.5 using default parameters and Diamond for orthologous searches (Buchfink et al., 2015; Huerta-Cepas et al., 2017); COGs terms and KEGG numbers and pathways were transferred by EggNOG mapper annotation; and InterproScan 5.31-70.0 (Jones et al., 2014) to detect functional domains based on sequence similarity and hidden Markov models (HMMs) searches. A scheme of the used in-house pipeline is provided as Supplemental Material (Supplementalry Figure 1).

### Comparative Analysis and Selection of Core Sequences

The conservation of all predicted genes was analyzed, and the gene repertoires of *P. salmonis* LF-89 and EM-90 genogroups were compared. The amino acid sequences were recovered in FASTA format for all genes predicted in the prediction step. These sequences were then grouped using CD-HIT stand-alone, version 4.8.1 (Fu et al., 2012) with 75% of sequence similarity and 50% coverage of both the largest and shortest sequences as a cutoff value to consider a given protein a member of a given cluster. A binary presence-absence matrix was constructed considering each gene cluster for each genogroup and obtained the core genes for each genogroup. These subsets were compared with the same parameters described previously (only the unique core genes from each genogroup were recovered). Finally, to corroborate the resulting comparisons, each gene was aligned against the opposite genogroup genome with GMAP version 2017-01-14 (Wu et al., 2016).

### Development and Optimization of a Multiplex PCR

Genes unique to each genogroup (LF-89 and EM-90) were annotated using the database of Clusters of Orthologous Groups of proteins (COGs) and EggNOG 4.5 (Huerta-Cepas et al., 2017). Evaluated sequences were annotated as “Non-Identified,” “Poorly Characterized” and removed those classified as “Information Storage and Processing.” Each sequence was manually curated using the BlastP tool against the *P. salmonis* database using 60% identity, 50% coverage, and E-value <10^–5^ (Ye et al., 2012). Primer sets were designed with the Primer-BLAST tool (Ye et al., 2012) using default parameters and the top gene/protein with functional annotations representative of each genogroup. Final amplicon sizes ranged between 100 and 200 nt. The annealing temperatures and efficiency of the reactions for each primer set were evaluated via conventional and quantitative PCR (qPCR), using chromosomal DNA from *P*. *salmonis* LF-89 EM-90 as templates. The primer sets with better experimental results in conventional PCRs for each genogroup were used for multiplex PCR. The reaction efficiency of the chosen primer sets was then evaluated by qPCR.

### Bacterial Cultures and Identification

All *P. salmonis* isolates analyzed in this study were grown using the SHK-1 cell line (a macrophage-like cell line from *S*. *salar* head kidney) for 10–15 days. The culture medium was centrifuged at 1,000 × *g* for 5 min at 18°C following the cytopathic effects. The supernatant was removed, the pellet was recovered, and grown up on Austral TS-HEM solid and in Austral SRS-broth culture media (Yañez et al., 2013; Yáñez et al., 2014). Culture purity was verified using Gram staining, IFAT, and nested PCRs (Mauel et al., 1996; Yañez et al., 2013).

### Bacterial DNA Extraction

Chromosomal DNA of the *P. salmonis* strains was extracted using the NucleoSpin Tissue Genomic DNA Purification Kit (Macherey-Nagel) as per the manufacturer’s recommendations. DNA quality was evaluated visually by agarose gel electrophoresis (0.8% wv^–1^ in TAE 1X buffer, SyberSafe, Invitrogen). DNA concentration was adjusted between 10 and 20 ng μL^–1^ with a ScanDrop analyzer (Analytik Jena) and stored samples at 4°C until further PCR amplification.

### PCRs and Visualization

DNA was amplified in a 25 μL reaction using the GoTaq Green Master Mix (Promega), following the manufacturer’s instructions. DNA fragments were amplified using a MaxyGene II Thermal Cycler (Axygen Scientific), and amplified products were detected on a 1.8% wv^–1^ agarose gel in TAE 1X buffer with SyberSafe using standard procedures. Real-time PCRs were conducted with GoTaq qPCR Master Mix (Promega) and a CFX96 Real-Time System (BIO-RAD).

### DNA Sequencing of 16S rRNA Genes

16S rRNA gene sequences were amplified using the 16SF and 16SR primer pair (Table 1) and a MaxyGene II Thermal Cycler (Axygen Scientific). The thermocycling protocol was as follows: initial denaturation at 95°C for 3 min, 35 cycles of amplification (denaturation at 95°C for 1 min, annealing at 45°C for 1 min and extension at 72°C for 1 min), and a final extension at 72°C for 5 min. The amplicons were visualized using agarose gel electrophoresis (1.8% wv^–1^ in TAE 1X buffer) and SyberSafe following standard procedures and used the E.Z.N.A Gel Extraction Kit (Omega-Biotek) to recover amplicons from agarose gels (according to the manufacturer’s instructions). DNA was stored at −20°C until further use. Macrogen (Korea) was used to sequence DNA samples using the dideoxynucleotide method (Sanger et al., 1977) and the above primer pair. All chromatograms were manually verified to ensure high sequencing quality results.

**TABLE 1:** List of primers used for genotyping *P. salmonis* isolates.

Manuscript code primers	Forward sequence	Reverse sequence	Product size (bp)	Accession number	Description
**LF-89 genogroup**					
660	GGCGCTGGGTTATTTTCACC	GTCCCTGTACTCATTCCGCC	110	ALB21840.1	MFS transporter
1755	ACACCTGTAGTTGCTGCTGG	GCAGCTTCAATGCCATTAGCC	131	WP_144420689.1	Nitronate monooxygenase
2701	ATGAAAATTTGCCTGCCTCACC	GTATGGCGGTATGCATCGGG	120	WP_027242646.1	Patatin-like phospholipase
**EM-90 genogroup**					
371	AAGCCATCCCCGTATACACC	TTACCAGTCGCTACTGAGCC	173	WP_016211797.1	Aldehyde dehydrogenase family protein
1207	TGACGAAGCGTATTGTTGCG	ACGCTATCGCCACATCATCC	177	WP_016210154.1	Acid phosphatase. class B-like
2825	GATTGATGAACAGCTTGCGGC	TGGTGATTCTTGGCCACTGC	175	WP_016210761.1	Tryptophan 2,3-dioxygenase
**Eubacterial 16S rRNA gene**					
16S Eubac	AGAGTTTGATCCTGGCTCAG	ACGGATACCTTGTTACGAGTT	∼1500		16S rDNA gene

### Phylogenetic Analysis

The genotyping methodology developed in the present study was verified using a phylogenetic tree constructed from 16S rRNA gene sequences of randomly selected *P. salmonis* isolates and by visualizing amplicons from the multiplex PCR on an agarose gel. The MEGA version X (Kumar et al., 2018) software was used to perform phylogenetic and molecular evolutionary analyses based on 16S rRNA gene sequences aligned with the DNA ClustalW algorithm using default parameters. An evolutionary molecular model was constructed using the option “Best D.N.A./Protein Model” from the “Model Selection” tool and phylogenetic reconstruction with the neighbor-joining tree algorithm with 1,000 bootstrap replicates. For this analysis, the 16S rRNA gene sequence was used of *Francisella noatunensis* as an outgroup.

## Results

### Comparative Genomics Identifies Unique Gene Candidates as Markers for EM and LF Genogroups

In this study, 73 fully sequenced *P. salmonis* genomes were retrieved from the NCBI database (Supplementary Table 1) and separated into LF-89, and EM-90 genogroups, based on hierarchical clustering of all ORFs predicted with the CD-Hit tool (Supplementary Figure 2). A total of 5,467 coding sequences were predicted, of which 2,653 and 2,814 were identified in LF-89 and EM-90 genomes, respectively.

Each genogroup’s sequences were separated into the core, and accessory coding sequences based on ORFs predicted for LF-89 and EM-90 genomes. *In silico* analysis of the LF-89 genogroup revealed 2,322 sequences shared between 35 genomes and showed that LF-89 genomes have 331 accessory coding sequences (Figure 1A) and the 38 EM-90 genomes shared 2,280 sequences in the core genome and had 534 accessory coding sequences (Figure 1B). A total of 1,801 clusters of shared gene sequences were found between LF-89 and EM-90 genogroups. LF-89 and EM-90 genogroups had 253 and 291 unique genes, respectively (Figure 1C).

Subsequently, 5,467 predicted coding sequences were functionally annotated using the COGs database from EggNOG 4.5 tool, and 158 and 250 sequences were annotated in LF-89 and EM-90 genogroups, respectively, after selecting the unique sequences in each genogroup (*E*-value annotation < 0.001).

**FIGURE 2:**
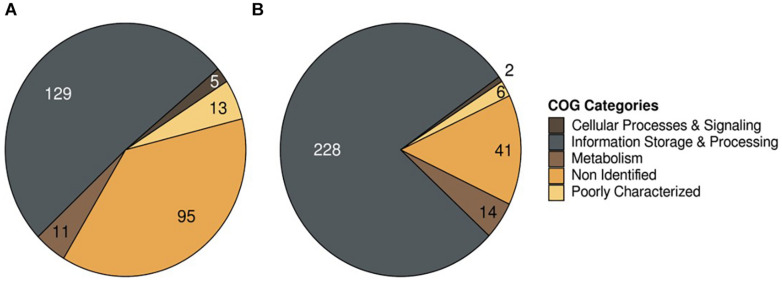
COG annotation of unique genes in *P. salmonis* genogroups. **(A)** LF-89 genogroup; **(B)** EM-90 genogroup.

Sequences were classified into three categories; Cellular Process & Signaling, Metabolism, and Poorly Characterized (Figure 2), and annotated the Poorly Characterized sequences in each dataset. Sequences annotated as Non-Identified, and Information storage and Processing were mobile elements (transposases and integrases), and therefore was removed from further analysis. Each selected sequence annotated by COG categories was manually curated against the *P. salmonis* database on NCBI using the BlastP tool to exclude sequences whose descriptions included “Hypothetical Protein” and “Unknown Function.” A total of 22 and 18 unique sequences were identified in the LF-89 and EM-90 genogroups, respectively (Supplementary Table 2). The characterization of each sequence group also indicated that 11 and 14 sequences were associated with “Metabolism” in LF-89 and EM-90 genetic groups, respectively. Moreover, three gene sequences of the LF-89 genogroup were related to “Cellular Processing and Signaling,” while only a sequence associated with this category was found in the EM-90 genogroup. The rest of the sequences were classified as “Function unknown” according to COG annotation in both genogroups.

Finally, six sequences were randomly selected (three for each genogroup) to design PCR primers with the Primer-Blast tool. Primers were designed such that amplicons were close to 130 bp in the LF-89 genogroup and approximately 170 bp in the EM-90 genogroup. The sequence was then manually analyzed using the BlastN tool to select the primer sets that specifically amplified the LF-89 or EM90 genogroup *in silico* (Table 1).

### Optimization of Genotyping PCR

**FIGURE 3:**
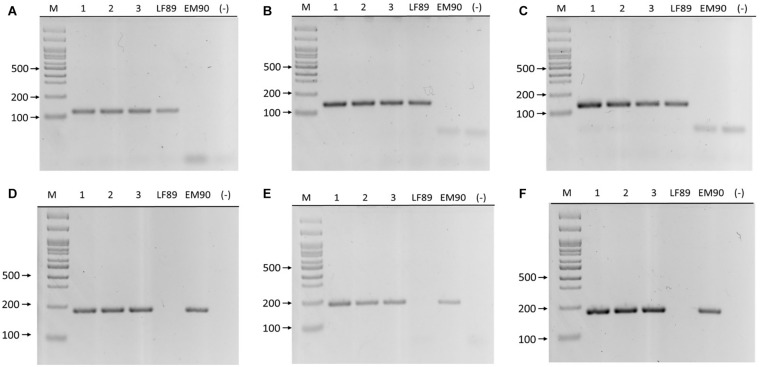
PCR amplification of LF-89 and EM-90 unique genes in *P. salmonis*. Upper panels **(A–C)** primer pairs 660, 1755, and 2701, respectively. Samples (1) AUS005, (2) AUS008, (3) AUS025, LF-89 strain, EM-90 like strain, and (–) negative control. Bottom panels **(D–F)** primer pairs 1207, 371, and 2825, respectively. Samples (1) AUS045, (2) AUS067, (3) AUS100, LF-89 strain, EM-90 like strain, and (–) negative control. (M) Molecular weight marker (100 bp Ladder).

The primer sets’ annealing temperatures for each genogroup were optimized by gradient PCR in separate reactions using genomic DNA from LF-89 (ATCC VR-1361) and EM-90 isolates as templates. Each set produced single amplicons at all temperatures in a range between 55 and 68°C (Table 1). The primers’ specificities were tested using DNA templates from their target and non-target genogroups using sequenced DNA samples, phylogenetic analysis of 16S rRNA gene sequences (unpublished data), and MLST for *P. salmonis*. Each primer set only amplified its target genogroup, and amplicons were ∼130 and ∼170 bp in the LF-89 and EM-90 samples. No other bands were observed on agarose gels (Figure 3).

**FIGURE 4:**
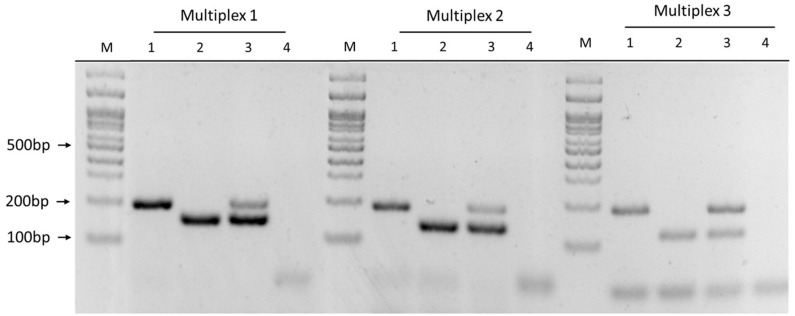
Multiplex PCR prototype using different primer mixes. Multiplex-1: 1755/1207 primer mix; Multiplex-2: 2701/371 primer mix; Multiplex-3: 660/1207 primer mix. Samples: (1) EM-90 strain, (2) LF-89 strain, (3) LF-89 and EM-90 like mix (1:1 ratio), (4) negative control, and (M) Molecular weight marker (100 bp Ladder).

The six primer sets were combined in nine Multiplex PCR prototypes (Multiplex-1 to Multiplex-9), evaluated their specificity with DNA from each genogroup, and discarded the prototypes with non-specific amplification by conventional PCR. The Multiplex-1 (1755/1207 primer mix), Multiplex-2 (2701/371 primer mix), and Multiplex-3 (660/1207 primer mix) prototypes were selected because they showed specific amplification of each genogroup (Figure 4) when PCR products were visualized via agarose gel electrophoresis. Three Multiplex PCR prototypes were used to identify both genogroups in a sample with a 1:1 ratio of DNA from both genogroups and observed two amplicons of the expected sizes (Figure 4, sample 3).

**FIGURE 5:**
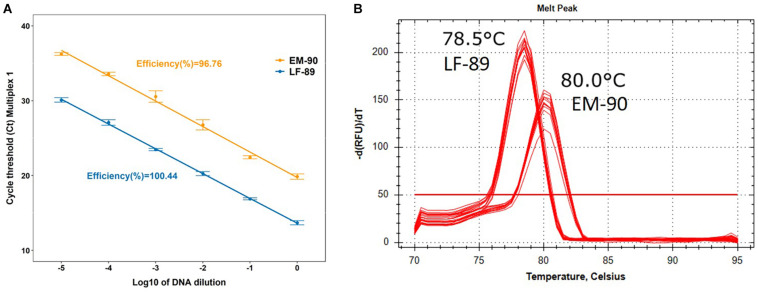
Characterization of Multiplex-1 prototype using qPCR. **(A)** Standard curve and efficiency of reaction using LF-89 and EM-90-like isolates as DNA templates. **(B)** Melting temperature of the amplicon from LF-89 and EM-90-like isolates.

The efficiency of each Multiplex PCR prototype was evaluated by absolute qPCR using the standard curve method. The Multiplex-1 prototype was 100.44 and 96.76% efficient with DNA of LF-89 and EM-90 isolates, respectively (Figure 5A); the Multiplex-2 prototype was 116.5 and 140.9% efficient, respectively, and the Multiplex-3 prototype was 118.4 and 93.2% efficient, respectively (Supplementary Figures 3A,B). Additionally, the melting temperatures of LF-89 and EM-90 PCR products from the Multiplex-1 prototype had a difference of 1.5°C (Figure 5B); however, they were the same for the Multiplex 2 and 3 prototypes (77.5 and 80°C, respectively) (Supplementary Figures 3A,B). The Multiplex-1 prototype was selected to validate our methodology using field samples because of the differences in melting temperatures between resulting amplicons from LF-89 and EM-90 DNA templates and the efficiencies of PCR reactions.

### Validation of Multiplex PCR

DNA was extracted from 48 *P. salmonis* isolates (kept in pure culture in our laboratory) with a NucleoSpin Tissue Genomic DNA Purification Kit and analyzed them using spectrophotometry. The samples were obtained from different hosts, years, and locations in Southern Chile (Table 2). Gram staining, IFAT, nested PCRs (data not shown) confirmed the purity of each culture.

**TABLE 2:** Genotyping results using multiplex PCR for 48 *P. salmonis* isolates obtained from different hosts (*Salmo salar, Oncorhynchus mykiss*, and *Oncorhynchus kisutch*), sources, years, and locations in Chile.

Isolation data							Typing data	
Isolate	Host	Source	Location	Year	16S rRNA gene	MLST Genotyping	LF-89	EM-90
AUS002	*O. kisutch*	Kidney	Castro	2008	Yes	LF-89-like	X	
AUS005	*O. mykiss*	Liver	Reloncavi	2008	Yes	LF-89-like	X	
AUS007	*O. kisutch*	Kidney	Castro	2008	Yes	LF-89-like	X	
AUS008	*O. mykiss*	Spleen	Fiordo Comau	N/D	Yes	LF-89-like	X	
AUS013	*S. salar*	Kidney	Huelmo	2010	Yes	LF-89-like	X	
AUS023	*S. salar*	Kidney	Castro – Chile	2008	Yes	LF-89-like	X	
AUS024	*S. salar*	Kidney	X – Region	2009	Yes	LF-89-like	X	
AUS025	*O. kisutch*	Kidney	Reloncavi	2010	Yes	LF-89-like	X	
AUS040	*S. salar*	Kidney	ND	2008	Yes	EM-90-like		X
AUS041	*O. mykiss*	Spleen	Reloncavi	2008	Yes	LF-89-like	X	
AUS042	*S. salar*	Kidney	Huelmo	2008	Yes	LF-89-like	X	
AUS043	*S. salar*	Kidney	Llancahue	2009	Yes	LF-89-like	X	
AUS044	*S. salar*	Kidney	Central - Chiloe	2010	Yes	LF-89-like	X	
AUS045	*O. mykiss*	Kidney	Punta Iglesias	2009	Yes	EM-90-like		X
AUS047	*S. salar*	Kidney	Huelmo	2008	Yes	EM-90-like		X
AUS050	*S. salar*	Kidney	Huelmo	2009	Yes	LF-89-like	X	
AUS055	*S. salar*	Kidney	Chiloe	2010	Yes	EM-90-like		X
AUS067	*S. salar*	Liver	Puerto Cisnes	2016	Yes	EM-90-like		X
AUS068	*S. salar*	Liver	Aysen	2016	Yes	EM-90-like		X
AUS070	*S. salar*	Spleen	Aysen	2016	Yes	EM-90-like		X
AUS071	*S. salar*	Liver	Puerto cisnes	2016	Yes	EM-90-like		X
AUS072	*S. salar*	Liver	Puerto Aguirre	2015	Yes	LF-89-like	X	
AUS073	*S. salar*	N/D	N/D	2016	Yes	LF-89-like	X	
AUS074	*S. salar*	Liver	N/D	2016	Yes	EM-90-like		X
AUS075	*O. kisutch*	External injury	N/D	2016	Yes	LF-89-like	X	
AUS077	*S. salar*	Liver	N/D	2016	Yes	EM-90-like		X
AUS078	*S. salar*	Kidney	N/D	2016	Yes	LF-89-like	X	
AUS079	*S. salar*	Brain	N/D	2015	Yes	EM-90-like		X
AUS080	*S. salar*	Kidney	N/D	2015	Yes	EM-90-like		X
AUS081	*S. salar*	Spleen	N/D	2015	Yes	EM-90-like		X
AUS082	*S. salar*	Liver	N/D	2015	Yes	EM-90-like		X
AUS083	*S. salar*	Liver	N/D	2015	Yes	LF-89-like	X	
AUS084	*S. salar*	Spleen	N/D	2016	Yes	LF-89-like	X	
AUS087	*O. mykiss*	Kidney	Central Chiloé	2015	Yes	LF-89-like	X	
AUS088	N/D	N/D	N/D	N/D	Yes	LF-89-like	X	
AUS089*	*O. kisutch*	Kidney	Puerto Montt	1989	Yes	LF-89-like	X	
AUS094	*O. mykiss*	Kidney	Antares	2016	Yes	LF-89-like	X	
AUS096	*O. mykiss*	Kidney	ALAO, Chiloé	2016	Yes	EM-90-like		X
AUS100	*S. salar*	Kidney	ALAO, Chiloé	2010	Yes	EM-90-like		X
AUS109	*S. salar*	Liver	Fiordo de Aysén	2017	Yes	EM-90-like		X
AUS112	*S. salar*	Kidney	Aysén	2015	Yes	EM-90-like		X
PS-005	*S. salar*	External injury	Aysén	2015	Yes	EM-90-like		X
PS-010	*S. salar*	Kidney	Aysén	2015	Yes	EM-90-like		X
PS-014	*S. salar*	Kidney	Aysén	2015	Yes	EM-90-like		X
PS-015	*S. salar*	Kidney	Aysén	2015	Yes	EM-90-like		X
MH-2	*S. salar*	Liver	Lilicura	2013	Yes	EM-90-like		X
MH-6	*S. salar*	Kidney	Central Chiloé	2013	Yes	EM-90-like		X
C1118	*O. kisutch*	Kidney	Central Chiloé	2013	Yes	EM-90-like		X

*LF89 strain, ATCC VR-1361. ND, non-data.

**FIGURE 6:**
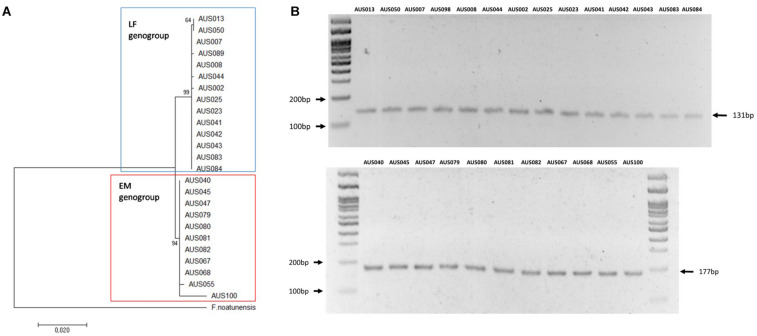
Comparative result of Multiplex-1 prototype and phylogenetic analysis of 16s rRNA gene sequences. **(A)** Molecular phylogenetic analysis using the maximum likelihood method. The Kimura 2-parameter model was used and Neighbor-Joining with 1,000 bootstraps. The 16S rRNA gene sequence of *F*. *noatunensis* was used as an outgroup. **(B)** Multiplex-1 prototype; upper and bottom panels depict isolates of LF-89 and EM-90 genogroups, respectively.

The samples were analyzed using the Multiplex-1 prototype by conventional PCR and visualized amplicons via agarose gel electrophoresis. These results were compared with genotyping using the MLST assay developed for *P. salmonis*, amplification, sequencing, and phylogenetic analysis of 16S rRNA genes. The same results were obtained with the three genotyping methodologies (Table 2 and Figure 6); 48% of isolates (*n* = 23) corresponded to the LF-89 genogroup, and the rest (*n* = 25) corresponded to the EM-90 genogroup. Genomic DNA was extracted from ten bacterial fish pathogens (donated by Ruben Avendaño-Herrera, Universidad Andres Bello) to evaluate the Multiplex-1 prototype specificity. The identification of each microorganism via amplification and sequencing of 16S rRNA genes was used as negative controls. No non-specific bands were observed with the Multiplex-1 prototype by conventional PCR (Supplementary Table 3) using DNA templates from non-target bacteria.

## Discussion

*Piscirickettsia salmonis* is the bacterium responsible for Piscirickettsiosis that affects seriously aquaculture worldwide, including the Chilean salmonid industry (Maisey et al., 2017). The disease has important economic impacts which has encouraged to many researchers to develop diverse strategies for the molecular characterization of the major clusters of strains associated to field disease outbreaks. In this sense, diverse studies based on the 16S rRNA gene analysis (sequencing, PCR-RFLP) (Mauel et al., 1999; Mandakovic et al., 2016; Aravena et al., 2020), phenotypic characterization (Otterlei et al., 2016), MLST (Isla et al., 2019) and comparative genomics (Bohle et al., 2014; Bravo and Martinez, 2016; Nourdin-Galindo et al., 2017) have indicated the existence of LF-89 and EM-90 genogroups containing epidemiologically relevant *P. salmonis* stains. Both genogroup isolates show differential susceptibility to florfenicol, oxytetracycline, and quinolones (Saavedra et al., 2017). Also, they have different culture temperatures, whereas the optimal growth temperature of LF-89 genogroups is 16 to18°C, EM-90 isolates grow best between 18 to 22°C (Saavedra et al., 2017). These genetic groups also cause different levels of morbidity during challenge assays; EM-90 isolates cause higher mortality over shorter periods than the LF-89 strain (Rozas-Serri et al., 2017, 2018). Additionally, EM-90 isolates are more pathogenic than LF-89 isolates and exhibit increased intracellular replication and decreased host survival rate (Rozas-Serri et al., 2017). These differences highlight the need to develop methodologies for rapid genotyping of *P. salmonis* in field samples.

Analysis of 73 genomes showed that *P*. *salmonis* could be segregated into two genogroups with 2,322 and 2,280 core sequences in LF-89 and EM-90 genogroups. The number of sequences was similar to that reported by Nourdin-Galindo et al. (2017), who reported core-genomes composed of 2,170 and 2,228 sequences for LF-89 and EM-90 genogroups respectively. Interestingly, different unique genes were identified than Nourdin-Galindo et al., especially in the case of the LF-89 genogroup. 253 and 291 unique sequences were identified in LF-89 and EM-90, whereas Nourdin-Galindo et al., identified 437 and 282 unique sequences in LF-89 and EM-90 clusters. These variations could be due to differences in the pipeline used to identify pan/core-genomes and unique genes. However, the most important factor in the decrease in the number of unique sequences identified in each genogroup may be the higher number of genomes analyzed. Thirty-five genomes of the LF-89 genogroup and 38 genomes of the EM-90 genogroup were analyzed in the present study compared to the 13 and 6 genomes analyzed by Nourdin-Galindo et al. (2017). Therefore, variability could be caused by the total number of genomes analyzed, so we highly recommend using at least five genomes for robust characterization of pan-genomes (Vernikos et al., 2015). Additionally, the core genome sizes in both genogroups were estimated to be similar to previous estimations using comparative genomics, where ∼8% of the bacterial genome was composed of core genes (Lapierre and Gogarten, 2009).

The description of the core/pan-genomes and unique genes or strain-specific sequences allowed us to identify molecular markers for phylogenetic analysis (Caputo et al., 2019). Three primer sets were designed to identify samples belonging to the LF-89 or EM-90 genogroup by conventional PCR by analyzing 73 complete *P. salmonis* genome sequences. A multiplex PCR for this classification was then validated by conventional and qPCR. Our taxonomic methodology for genotyping differs from classical approximations based on polymorphisms of 16S rRNA gene sequences, where primer sets are designed for small variable regions of these genes (Mauel et al., 1996). We recommend using the 16S rRNA gene to establish phylogenetic affiliations; however, this gene is highly conserved; therefore, it is not ideal for distinguishing closely related microorganisms (Rosselló-Mora and Amann, 2001).

Regarding the characterization of the 16S rDNA gene of *P. salmonis*, initial research showed that the sequences have high similarity (98.5%) with a difference of 22 nucleotides between the LF-89 and EM-90 genogroups (Mauel et al., 1999). The 16S rDNA analysis requires DNA sequencing, which is costly and time-consuming. Therefore, there is a need for new genotyping strategies for microorganisms. Others have proposed methods based on PCR-RLFP (Aravena et al., 2020) and MLST (Isla et al., 2019); however, these methodologies are qualitative and only indicate the presence or absence of the pathogen in a sample. Previous reports have used relative qPCR to measure the growth of *P. salmonis* in different tissues by amplification of 16S rRNA genes, Rozas-Serri et al. (2018) showed the importance of quantifying the load and growth of pathogens in post-smolt salmon infected with LF-89 or EM-90 strains (Rozas-Serri et al., 2018). They were able to quantify the growth of each strain by identifying and amplifying genes encoding conserved cellular functions in the core genome of each genogroup.

Our multiplex method generates amplicons with different melting temperatures and allows fast identification of the sample’s genogroup. The strain-mixed described by Isla et al., 2019 was omitted because there were very few whole-genome sequences in the database, so the core-genome of those strains could not be determined.

The Multiplex-1 prototype was used for analyzing DNA from other bacterial fish pathogens and showed that our primer sets specifically amplify genes unique to *P*. *salmonis* LF-89 and EM-90 genogroups. Additionally, our genotyping results of *P. salmonis* samples clustered into the two expected genogroups. The distribution of samples was also compared with the previous characterization of the same samples using the MLST developed for *P. salmonis* (Isla et al., 2019) or phylogenetic analysis of 16S rRNA gene sequences. The same dichotomous distribution of samples was observed using both methodologies and found both genogroups are widely distributed among the different fish hosts. This finding differs from previous reports reporting that EM-90 genogroups are responsible for most Piscirickettsiosis cases (Saavedra et al., 2017; Aravena et al., 2020). Therefore, we recommend using a higher number of field isolates and epidemiological studies to take different hosts into account.

In the present study, Bioinformatic analysis of fully sequenced *P. salmonis* genomes was used to design specific PCR primers against LF-89 and EM-90 genogroups to construct a novel diagnostic multiplex PCR tool. The multiplex-1 prototype is rapid, precise, and reliable and will reduce diagnosis times and costs. It will be helpful in routine veterinary diagnoses when using fish samples directly or pure and mixed bacterial cultures.

## Data Availability Statement

The dataset analyzed for this study can be found in Genbank (https://www.ncbi.nlm.nih.gov/genbank/), and accession numbers can be found in the article/Supplementary Material (Supplementary Table 1).

## Author Contributions

AY and VM-C: conceptualization. JF, AY, and AI: funding acquisition. AY and MR: project administration. AI, JM-H, and DH: methodology, sampling, and analysis. AI, VM-C, and JM-H: writing—original draft. AY and HL: writing—review and editing. All authors contributed to the article and approved the submitted version.

## Conflict of Interest

The authors declare that the research was conducted in the absence of any commercial or financial relationships that could be construed as a potential conflict of interest. The reviewer, DT, declared a shared affiliation, with the authors, JM-H and VM-C, to the handling editor at the time of the review.
