# Supplementary material for: Development of a Multiplex PCR Assay for Genotyping the Fish Pathogen Piscirickettsia salmonis Through Comparative Genomics
Source: Front Microbiol. 2021 Jun 11;12:673216. doi: 10.3389/fmicb.2021.673216 (PMC8226252; doi:10.3389/fmicb.2021.673216)
Supplement: Supplementary file 1 [file Table_1.DOCX]

Supplementary Material

## Supplementary Figures


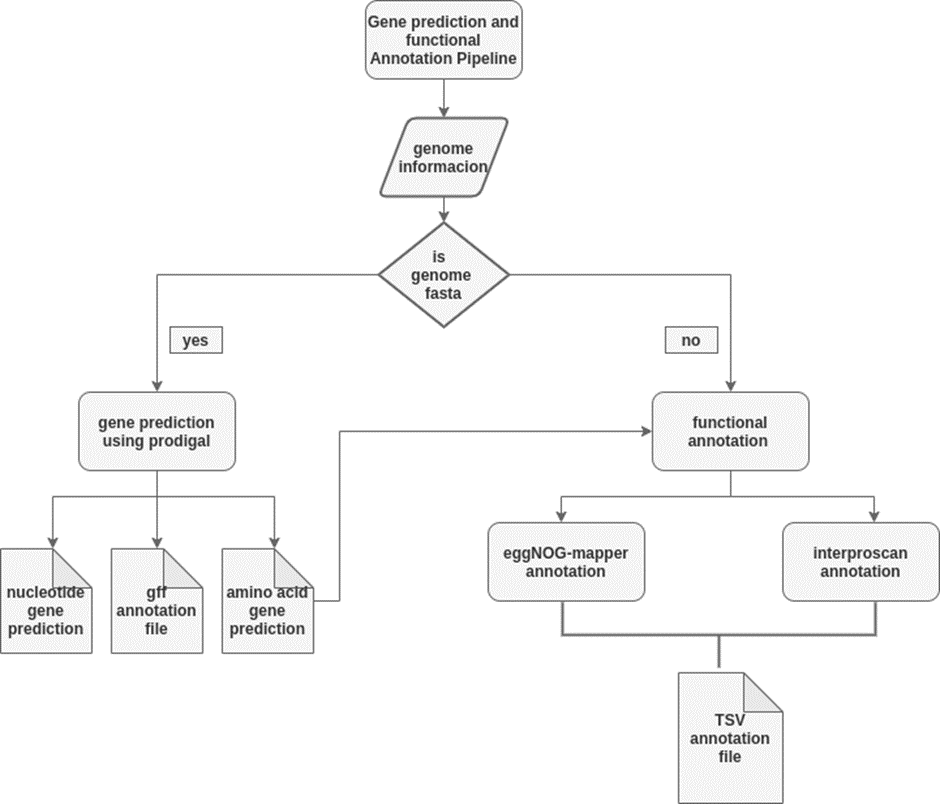


Supplementary Figure 1. Scheme of the in-house pipeline for prediction of protein-encoding genes in P. salmonis genomes.


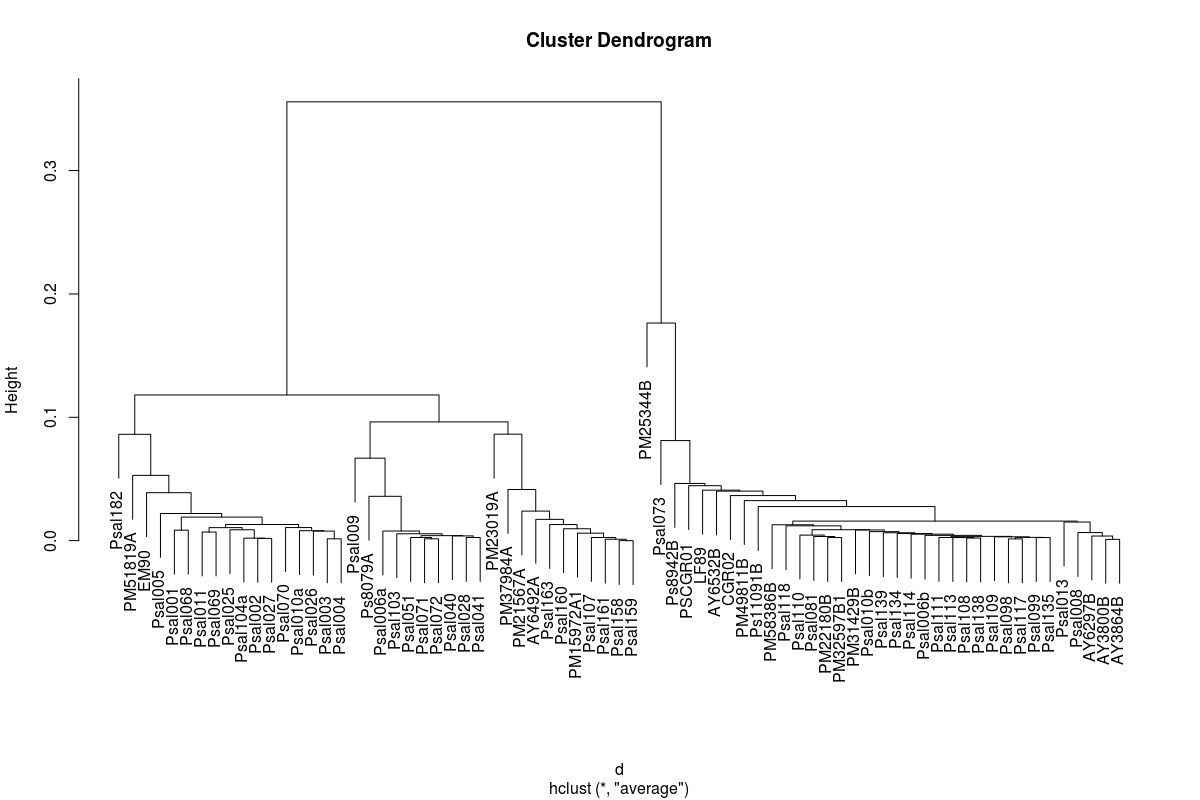


Supplementary Figure 2. Clustering of P. salmonis genomes. Analysis based in open read frames prediction development in CD-Hit tool.


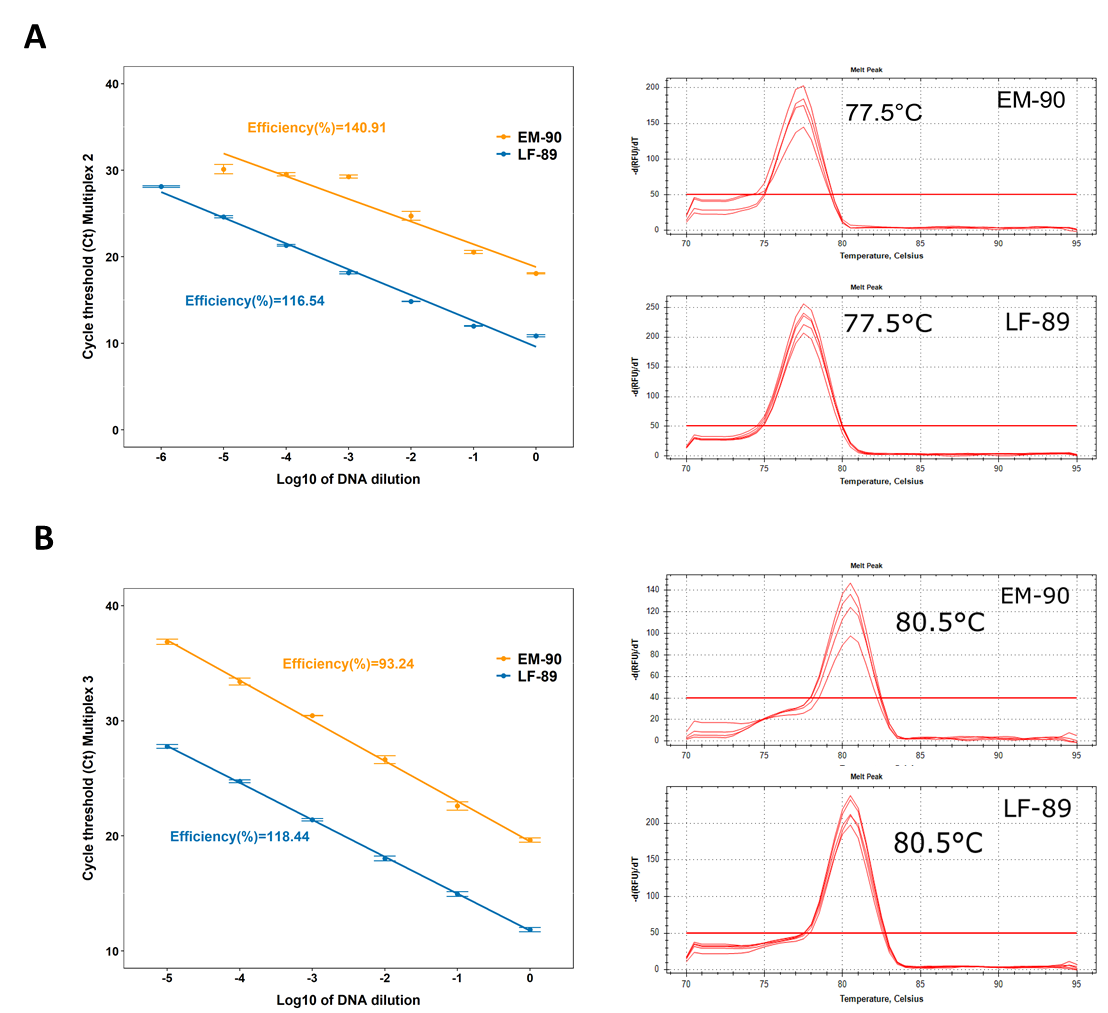


**Supplementary Figure 3. Determination of efficiency of reaction and melting temperature.** A) Multiplex-2 prototype (2701/371) and B) Multiplex-3 prototype (660/1207). Samples DNA of LF-89 and EM-90 like strains.

## Supplementary Tables

**Supplementary Table 1**. General description of fully sequenced *Piscirickettsia salmonis* genomes available at the NCBI database**.** All information was accessed in September 2020

| Accession number | Strain | Length (Mb) | GC content (%) | Host |
| --- | --- | --- | --- | --- |
| CP011849.2 | LF-89 ATCC VR-1361 | 3.51 | 39.62 | *Oncorhynchus kisutch* |
| CP039240.1 | MR5 | 4.15 | 39.73 | *Salmo salar* |
| CP039227.1 | SR1 | 4.15 | 39.74 | *Salmo salar* |
| CP039234.1 | BI1 | 4.07 | 39.72 | *Salmo salar* |
| CP038908.1 | Psal-009 | 3.56 | 39.64 | *Oncorhynchus kisutch* |
| CP039219.1 | NVI 5692 | 3.76 | 39.67 | *Salmo salar* |
| CP050938.1 | Ps-2192A | 3.70 | 39.60 | *Salmo salar* |
| CP038927.1 | Psal-013 | 3.49 | 39.67 | *Salmo salar* |
| CP048056.1 | Ps-8942B | 3.71 | 39.65 | *Salmo salar* |
| CP013975.1 | CGR02 | 3.42 | 39.64 | *Salmo salar* |
| CP038904.1 | Psal-008 | 3.45 | 39.67 | *Oncorhynchus mykiss* |
| CP038898.1 | Psal-006b | 3.60 | 39.65 | *Salmo salar* |
| CP039060.1 | Psal-099 | 3.51 | 39.62 | *Oncorhynchus mykiss* |
| CP038918.1 | Psal-010b | 3.51 | 39.63 | *Oncorhynchus kisutch* |
| CP039055.1 | Psal-098 | 3.51 | 39.62 | *Oncorhynchus mykiss* |
| CP039112.1 | Psal-135 | 3.52 | 39.63 | *Oncorhynchus mykiss* |
| CP039087.1 | Psal-111 | 3.51 | 39.62 | *Oncorhynchus kisutch* |
| CP039107.1 | Psal-134 | 3.52 | 39.63 | *Oncorhynchus mykiss* |
| CP039097.1 | Psal-117 | 3.50 | 39.62 | *Oncorhynchus kisutch* |
| CP039171.1 | Psal-138 | 3.51 | 39.62 | *Oncorhynchus mykiss* |
| CP039868.1 | Psal-113 | 3.50 | 39.62 | *Oncorhynchus kisutch* |
| CP039076.1 | Psal-109 | 3.51 | 39.62 | *Oncorhynchus kisutch* |
| CP039176.1 | Psal-139 | 3.51 | 39.62 | *Oncorhynchus mykiss* |
| CP039070.1 | Psal-108 | 3.53 | 39.62 | *Oncorhynchus kisutch* |
| CP039092.1 | Psal-114 | 3.51 | 39.62 | *Oncorhynchus kisutch* |
| CP048052.1 | Ps-11091B | 3.52 | 39.65 | *Oncorhynchus kisutch* |
| CP013786.1 | PM58386B | 3.52 | 39.62 | *Salmo salar* |
| CP039102.1 | Psal-118 | 3.51 | 39.62 | *Oncorhynchus kisutch* |
| CP013806.1 | PM31429B | 3.52 | 39.62 | *Oncorhynchus mykiss* |
| CP013781.1 | PM49811B | 3.52 | 39.62 | *Salmo salar* |
| CP039081.1 | Psal-110 | 3.52 | 39.62 | *Oncorhynchus kisutch* |
| CP039046.1 | Psal-073 | 3.55 | 39.65 | *Salmo salar* |
| CP013811.1 | AY3864B | 3.52 | 39.83 | *Salmo salar* |
| CP013791.1 | AY6297B | 3.52 | 39.83 | *Salmo salar* |
| CP013796.1 | AY6532B | 3.49 | 39.71 | *Salmo salar* |
| CP039050.1 | Psal-081 | 3.51 | 39.62 | *Oncorhynchus mykiss* |
| CP013801.1 | PM22180B | 3.51 | 39.62 | *Oncorhynchus mykiss* |
| CP012508.1 | PM32597B1 | 3.51 | 39.62 | *Oncorhynchus kisutch* |
| CP033937.1 | EM-90 | 3.69 | 39.61 | *Salmo salar* |
| CP061189.1 | Ps12201A | 3.65 | 39.56 | *Salmo salar* |
| CP048066.1 | Ps-8079A | 3.53 | 39.74 | *Salmo salar* |
| CP013944.1 | PSCGR01 | 3.49 | 39.62 | *Oncorhynchus mykiss* |
| CP038891.1 | Psal-005 | 3.32 | 39.67 | *Salmo salar* |
| CP039032.1 | Psal-070 | 3.35 | 39.65 | *Salmo salar* |
| CP039214.1 | Psal-104a | 3.45 | 39.61 | *Salmo salar* |
| CP038942.1 | Psal-027 | 3.45 | 39.61 | *Salmo salar* |
| CP038952.1 | Psal-040 | 3.48 | 39.74 | *Salmo salar* |
| CP038932.1 | Psal-025 | 3.52 | 39.57 | *Oncorhynchus mykiss* |
| CP039035.1 | Psal-071 | 3.45 | 39.65 | *Salmo salar* |
| CP038923.1 | Psal-011 | 3.42 | 39.63 | *Salmo salar* |
| CP038913.1 | Psal-010a | 3.40 | 39.61 | *Oncorhynchus kisutch* |
| CP038957.1 | Psal-041 | 3.43 | 39.66 | *Salmo salar* |
| CP038947.1 | Psal-028 | 3.47 | 39.74 | *Salmo salar* |
| CP038893.1 | Psal-006a | 3.47 | 39.65 | *Salmo salar* |
| CP039209.1 | Psal-103 | 3.44 | 39.65 | *Salmo salar* |
| CP038876.1 | Psal-002 | 3.43 | 39.61 | *Salmo salar* |
| CP038962.1 | Psal-051 | 3.45 | 39.74 | *Salmo salar* |
| CP038937.1 | Psal-026 | 3.39 | 39.61 | *Salmo salar* |
| CP013778.1 | PM51819A | 3.43 | 39.64 | *Salmo salar* |
| CP039040.1 | Psal-072 | 3.71 | 39.71 | *Salmo salar* |
| CP038972.1 | Psal-069 | 3.38 | 39.65 | *Salmo salar* |
| CP038881.1 | Psal-003 | 3.38 | 39.61 | *Oncorhynchus kisutch* |
| CP038886.1 | Psal-004 | 3.38 | 39.61 | *Salmo salar* |
| CP039204.1 | Psal-182 | 3.39 | 39.61 | *Salmo salar* |
| CP038811.1 | Psal-001 | 3.41 | 39.70 | *Oncorhynchus kisutch* |
| CP038967.1 | Psal-068 | 3.40 | 39.70 | *Salmo salar* |
| CP012413.1 | PM15972A1 | 3.25 | 39.73 | *Salmo salar* |
| CP039201.1 | Psal-163 | 3.30 | 39.73 | *Salmo salar* |
| CP039190.1 | Psal-160 | 3.34 | 39.72 | *Salmo salar* |
| CP013768.1 | PM23019A | 3.31 | 39.64 | *Salmo salar* |
| CP039186.1 | Psal-159 | 3.33 | 39.64 | *Salmo salar* |
| CP039181.1 | Psal-158 | 3.33 | 39.63 | *Salmo salar* |
| CP039065.1 | Psal-107 | 3.36 | 39.62 | *Oncorhynchus mykiss* |
| CP039195.1 | Psal-161 | 3.40 | 39.62 | *Salmo salar* |
| CP013773.1 | PM37984A | 3.29 | 39.65 | *Salmo salar* |
| CP013762.1 | PM21567A | 3.35 | 39.62 | *Salmo salar* |
| CP013757.1 | AY6492A | 3.35 | 39.62 | *Salmo salar* |
| CP013821.1 | PM25344B | 3.51 | 39.62 | *Oncorhynchus mykiss* |

**Supplementary Table 2.** List of unique sequences of LF-89 and EM-90 genogroups associated with Cellular Process and Signaling, Metabolism and Poorly Characterized according to COG classification using EggNOG 4.5

| **LF-89 genogroup** | | | | | **EM-90 genogroup** | | | | |
| --- | --- | --- | --- | --- | --- | --- | --- | --- | --- |
| **Code** | **Accession number** | **Description** | **COG class** | **E-value COG** | **Code** | **Accession number** | **Description** | **COG class** | **E-value COG** |
| 114 | WP_027242700 | Glycosyltransferase family 4 protein | M | 6.0E-79 | 1508 | WP_016212275.1 | MFS transporter | P | 4.6E-60 |
| 115 | WP_027242699.1 | Glycosyltransferase family 4 protein | M | 9.9E-147 | 1512 | WP_016212589.1 | MFS transporter | EGP | 5.9E-15 |
| 1699 | WP_017377202.1 | Short chain dehydrogenase | IQ | 3.0E-62 | 216 | WP_016209855.1 | VOC family protein | E | 2E-30 |
| 2086 | WP_027243102.1 | STAS domain-containing protein | T | 7.5E-09 | 2328 | WP_016211000.1 | Class I SAM-dependent methyltransferase | Q | 3E-54 |
| 262 | WP_027243085.1 | YbfB/YjiJ family MFS transporter | EGP | 5.2E-24 | 2368 | WP_016210195.1 | Cytidine deaminase | F | 3.4E-25 |
| 2639 | WP_017377116.1 | Copper chaperone PCu(A)C | P | 2.9E-10 | 2423 | WP_032126712.1 | MFS transporter | EGP | 3.2E-19 |
| 2642 | WP_017377119.1 | LysE family translocator | E | 2.4E-49 | 2479 | WP_016212048.1 | Disulfide bond formation protein B | C | 2.6E-36 |
| 2688 | WP_017376863.1 | MFS transporter | EGP | 5.3E-130 | 2587 | WP_032126712.1 | MFS transporter | EGP | 3.2E-19 |
| 2722 | WP_027242656.1 | Ion transporter | P | 5.1E-36 | 2825 | WP_016210761.1 | Tryptophan 2,3-dioxygenase | E | 1.1E-79 |
| 660 | ALB21840.1 | MFS transporter | EGP | 1.3E-52 | 366 | WP_036794771.1 | APC family permease | E | 2.4E-24 |
| 661 | WP_196426498.1 | Aminotransferase class I/II-fold pyridoxal phosphate-dependent enzyme | E | 1.6E-74 | 369 | WP_016211794.1 | Acyl-CoA/acyl-ACP dehydrogenase | I | 6.8E-28 |
| 662 | WP_017377737.1 | Inosine/xanthosine triphosphatase | F | 8.0E-54 | 371 | WP_016211797.1 | Aldehyde dehydrogenase family protein | C | 5E-116 |
| 2701 | WP_027242646.1 | Patatin-like phospholipase | S | 2.4E-47 | 420 | WP_016212552.1 | O-methyltransferase family protein | Q | 7.1E-24 |
| 1755 | WP_144420689.1 | Nitronate monooxygenase | S | 2.1E-44 | 424 | WP_016210211.1 | O-methyltransferase | Q | 4.9E-28 |
| 1271 | WP_196426495.1 | DotU family type IV/VI secretion system protein | S | 1.2E-11 | 516 | WP_016212477.1 | Type II toxin-antitoxin system Phd/YefM family antitoxin | D | 2.9E-27 |
| 2163 | WP_027242872.1 | SUMF1/EgtB/PvdO family nonheme iron enzyme | S | 1.7E-29 | 1207 | WP_016210154.1 | Acid phosphatase. class B-like | S | 5.0E-61 |
| 2375 | WP_036774583.1 | LexA family transcriptional regulator | S | 2.6E-19 | 515 | WP_032126794.1 | PINc domain-containing protein | S | 2.8E-40 |
| 2640 | WP_026063598.1 | Amidohydrolase family protein | S | 1.7E-50 | 1519 | WP_016210839.1 | Alpha/beta hydrolase | S | 1.3E-49 |
| 2712 | WP_017376888.1 | DotD/TraH family lipoprotein | S | 1.6E-16 |  |  |  |  |  |
| 2721 | WP_155046566.1 | DedA family protein | S | 7.9E-15 |  |  |  |  |  |
| 648 | WP_017377194.1 | Ergothioneine biosynthesis protein EgtB | S | 3.5E-111 |  |  |  |  |  |
| 649 | WP_048875913.1 | L-histidine N(alpha)-methyltransferase | S | 4.6E-63 |  |  |  |  |  |

**Supplementary Table 3.** Non-target Bacteria used in multiplex PCR assays.

| Isolate/ Type strain^(t)^ | 16S rDNA | PCR result |
| --- | --- | --- |
| *Flavobacterium psychrophilum* NCIMB 1947^t^ | Yes | Non-specific band |
| *Vibrio toranzoniae* CECT 7225^t^ | Yes | Non-specific band |
| *Tenacibaculum maritimum* CECT 4276 | Yes | Non-specific band |
| *Tenacibaculum dicentrarchi*  TdCh05 | Yes | Non-specific band |
| *Vibrio ordalii* Vo-18-LM | Yes | Non-specific band |
| *Vibrio anguillarum* ATCC 43307 | Yes | Non-specific band |
| *Vibrio tapetis* subsp. *quintayensis* Q050 | Yes | Non-specific band |
| *Flavobacterium columnare* ATCC UNAB | Yes | Non-specific band |
| *Streptococcus phocae* ATCC Soraya | Yes | Non-specific band |
| *Yersinia ruckeri* CECT 955 | Yes | Non-specific band |
